# Organic photovoltaics for simultaneous energy harvesting and high-speed MIMO optical wireless communications

**DOI:** 10.1038/s41377-021-00487-9

**Published:** 2021-02-23

**Authors:** Iman Tavakkolnia, Lethy K. Jagadamma, Rui Bian, Pavlos P. Manousiadis, Stefan Videv, Graham A. Turnbull, Ifor D. W. Samuel, Harald Haas

**Affiliations:** 1 LiFi Research and Development Centre, Department of Electronic & Electrical Engineering, The University of Strathclyde, Technology & Innovation Centre, 99 George Street, Glasgow, G1 1RD UK; 2 Organic Semiconductor Centre, SUPA, School of Physics and Astronomy, University of St Andrews, St Andrews, KY16 9SS UK; 3 pureLiFi, Rosebery House, 9 Haymarket Terrace, Edinburgh, EH12 5EZ UK

**Keywords:** Solar energy and photovoltaic technology, Optoelectronic devices and components, Fibre optics and optical communications

## Abstract

We show that organic photovoltaics (OPVs) are suitable for high-speed optical wireless data receivers that can also harvest power. In addition, these OPVs are of particular interest for indoor applications, as their bandgap is larger than that of silicon, leading to better matching to the spectrum of artificial light. By selecting a suitable combination of a narrow bandgap donor polymer and a nonfullerene acceptor, stable OPVs are fabricated with a power conversion efficiency of 8.8% under 1 Sun and 14% under indoor lighting conditions. In an optical wireless communication experiment, a data rate of 363 Mb/s and a simultaneous harvested power of 10.9 mW are achieved in a 4-by-4 multiple-input multiple-output (MIMO) setup that consists of four laser diodes, each transmitting 56 mW optical power and four OPV cells on a single panel as receivers at a distance of 40 cm. This result is the highest reported data rate using OPVs as data receivers and energy harvesters. This finding may be relevant to future mobile communication applications because it enables enhanced wireless data communication performance while prolonging the battery life in a mobile device.

## Introduction

Wireless data access is a necessity in today’s data-driven world, which affects all facets of modern societies, including health, commerce, politics, and education. The demand for mobile data has been growing at a rate of 60% annually over the past 13 years^1^, and there is no evidence to suggest that this exponential growth rate will slow down in the foreseeable future. In contrast, emerging new technologies, such as ultrahigh-definition video streaming, online virtual reality, online gaming, cloud-based services and the Internet of Things (IoT), require unprecedented data rates. It is reported in ref. ^2^ that the IoT is expected to connect 500 billion smart devices by 2030. Currently, wireless access is essentially realized using the radio frequency (RF) spectrum. However, recent predictions made from available data and assumptions based on generally accepted forecasts show^3^ that there will not be a sufficient new RF spectrum for cellular communications by 2035. Therefore, there is a need to explore other parts of the electromagnetic spectrum for wireless data access to combat the RF spectrum crunch. In this context, optical wireless communication (OWC) is of particular interest because it uses a vast and unregulated optical spectrum^4^. In particular, Light-Fidelity (LiFi), which is defined as a bidirectional, high-speed and fully networked OWC system, is a proven solution for mobile wireless networking^5^. Currently, there are global efforts to develop LiFi for future generations of wireless communication systems. To this end, LiFi is being standardized within the IEEE 802.11bb task group. The same standardization body, IEEE 802.11, has very successfully standardized Wi-Fi. Digital data encoding in OWC systems is based on intensity modulation (IM) of light sources such as lasers or light-emitting diodes (LEDs). Consequently, data decoding is based on direct detection (DD) by one or more photodetectors (PDs). Different kinds of light sources and PDs have been used for OWC in a wide range of applications^6–11^. For instance, data rates as high as 15.7 Gb/s have been demonstrated by the efficient utilization of inexpensive off-the-shelf LEDs and high-speed silicon PDs^12^.

Solar cells offer significant promise as high-speed data receivers, in addition to their main usage as energy-harvesting devices, as previously demonstrated in ref. ^13,14^, and more recently, data rates of up to 500 Mb/s from a single gallium arsenide (GaAs) solar cell have been reported by Fakidis et al.^7^. This dual function of photovoltaic (PV) systems is beneficially exploited for a wide variety of applications ranging from self-powered long-range free-space optical systems, where a large receiver exhibits significant advantages, to self-powered wearable devices as part of the future IoT^15^. Inorganic PVs, such as silicon (Si) and GaAs, exhibit high power conversion efficiencies (PCEs) (>25%) under solar spectrum illumination (air mass (AM) 1.5 G, corresponding to 100 mW/cm^2^). However, their use as optical receivers under indoor illumination is less efficient because the bandgaps of Si and GaAs (1.1 and 1.4 eV, respectively) are not well matched to the spectra of indoor lighting sources such as white LEDs and fluorescent lamps. This nonoptimal spectral overlap leads to a considerable drop in the PCE under indoor lighting conditions owing to carrier thermalization^16,17^. On the other hand, organic photovoltaics (OPVs) with conjugated organic semiconductors as the light-absorbing photoactive layer have an excellent spectral overlap with indoor artificial light. This leads to a 2–3 times enhanced PCE for OPVs compared with silicon solar cells, for which the efficiency drops (by two thirds) under indoor lighting^17,18^. Furthermore, OPVs are bandgap tuneable, printable, lightweight, flexible and amenable to roll-to-roll processing on customized and non-customized substrates^19,20^. Therefore, powering a large portion of connected mobile IoT devices using flexible OPVs is an attractive solution^21^ to overcome the burden of having to charge devices regularly.

Apart from the recently reported 500 Mb/s data rate with GaAs inorganic PVs^7^, other works are mainly based on commercial silicon PVs with data rates below 20 Mb/s^22–24^. For instance, a solar panel model for simultaneous energy harvesting and data transmission was analyzed by Wang et al.^14^, and a data rate of 11.84 Mb/s was reported while harvesting ~2 mW of electrical power. A self-reverse-biased solar panel optical receiver was presented by Shin et al.^25^, showing a data rate of 17.05 Mb/s. The promising potential of OPVs has also been previously demonstrated for two active layer blend structures of P3HT:PC_61_BM [poly(3-hexylthiophene) (P3HT) and phenyl C61-butyric acid methyl ester] and PTB7:PC_71_BM (poly[[4,8-bis[(2-ethylhexyl)oxy]benzo[1,2-b:4,5-b′]dithiophene-2,6-diyl][3-fluoro-2-[(2-ethylhexyl)carbonyl]thieno[3,4-b]thiophenediyl]] (PTB7):[6,6]-phenylC_71_-butyric acid methyl ester(PC_71_BM)) with data rates up to 50 Mb/s^26,27^. However, in each of these reports, the OPV device configuration was ITO/PEDOT:PSS/active layer/Ca or LiF/Al, where PEDOT:PSS (poly(3,4-ethylenedioxythiophene)-poly(styrenesulfonate)) is the hole extraction layer and calcium (Ca) or lithium fluoride (LiF) functions as the electron extraction layer. This standard structure suffers from severe performance instability and degradation issues accelerated by the inherent acidic and hygroscopic nature of PEDOT:PSS and the fast oxidation of low-work-function electrodes such as calcium and aluminum^28^. However, performance stability is a crucial factor required for integrating OPVs into future applications. Previous studies have shown that an inverted device architecture, in which the polarity of charge collection is reversed, with metal oxide-based charge-selective transport layers and higher-work-function metal electrodes, such as gold/silver (Au/Ag), has better performance stability than the standard structure^29^. Hence, in this paper, OPVs with inverted device architectures are explored along with judiciously selected organic photoactive blend systems to combine a high data rate transmission capability with the known performance stability of the inverted structure. These types of OPVs have not been previously explored for OWC.

Multiple individual small OPVs can be incorporated into a single solar panel to harvest energy (a few milliwatts) for various applications. Furthermore, multiple light sources are usually installed in an indoor environment, and several LEDs are implemented in a single illumination device. This facilitates designing a multiple-input multiple-output (MIMO) structure based on OPVs. MIMO communication systems have been developed in theory and through experiments for several OWC applications^30^. The merits and limitations of different techniques have been investigated^31^. An important benefit of an OPV-based MIMO system is that several independent data streams can be multiplexed to increase the overall data transmission rate while harvesting the aggregate energy of all individual solar cells. In addition, there is no need for electronics in the receiver to provide a negative bias voltage, and since the output is voltage, a transimpedance amplifier is not required. This may lead to simpler receiver circuitry. In fact, the analog electronics may only comprise passive electrical components^14^. The advantages especially unfold in an OPV-based MIMO system because it requires multiple receiver chains. Therefore, this novel system architecture can lead to an efficient solution for high data rate communication for future smart device applications. Note that the challenge in developing a MIMO–OWC system is interchannel cross-talk, which may limit the overall achievable data rate, and thus, cross-talk compensation methods may be necessary.

**Fig. 1:**
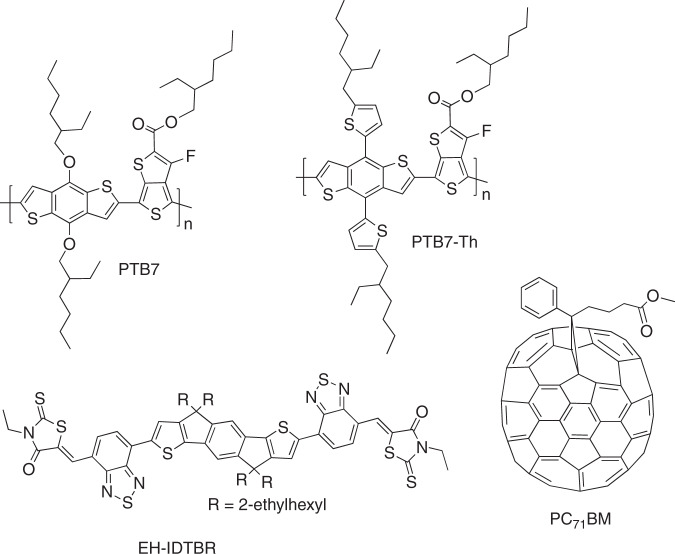
Molecular structures of donor and acceptor molecules. PTB7 stands for the donor (poly[[4,8-bis[(2-ethylhexyl)oxy]benzo[1,2-b:4,5-b′]dithiophene-2,6-diyl][3-fluoro-2-[(2-ethylhexyl)carbonyl]thieno[3,4-b]thiophenediyl]]. PTB7-Th corresponds to the donor (poly[4,8-bis(5-(2-ethylhexyl)thiophen-2-yl)benzo[1,2-b4,5-b']dithiophene-2,6-diyl-alt-(4-(2-ethylhexyl)-3-fluorothieno [3,4-b]thiophene-)-2-carboxylate-2-6-diyl]). PC_71_BM stands for the acceptor [6,6]-phenylC_71_-butyric acid methyl ester. EH-IDTBR corresponds to the acceptor 5,5'-[[4,4,9,9-tetrakis(2-ethylhexyl)-4,9-dihydro-s-indaceno[1,2-b:5,6-b']dithiophene-2,7-diyl]bis(2,1,3-benzothiadiazole-7,4-diylmethylidyne)]bis[3-ethyl-2-thioxo-4-thiazolidinone].

In this paper, we demonstrate a high bandwidth (i.e., 2.8 MHz) and record communication rates (i.e., 363 Mb/s) from stable inverted OPVs. The organic photoactive layer is composed of a bulk heterojunction (BHJ) of the highly efficient polymer donor PTB7-Th (poly[4,8-bis(5-(2-ethylhexyl)thiophen-2-yl)benzo[1,2-b4,5-b’]dithiophene-2,6-diyl-alt-(4-(2-ethylhexyl)-3-fluorothieno [3,4-b]thiophene-)-2-carboxylate-2–6-diyl]) with fullerene and nonfullerene acceptors (PC_71_BM and EH-IDTBR), as shown in Fig. 1. The size of each OPV cell is 4 mm by 2.5 mm. Under 1 Sun intensity (100 mW/cm^2^), these solar cells demonstrate a PCE of 8.8%, and under artificial indoor low-light-intensity illumination (0.3–6 mW/cm^2^), the PCE is 14%. To validate and assess the communication capabilities of these OPVs, a laser-based point-to-point OWC system is developed, and orthogonal frequency division multiplexing (OFDM) is incorporated to efficiently maximize the achievable data rate. The transmitted power of each laser is 56.2 mW. A data rate of 147.5 Mb/s is achieved with a single-input single-output (SISO) OPV-based system, which is the highest reported data rate with OPVs to the best of the authors’ knowledge. In addition, 3.7 mW is simultaneously harvested by the same system. These values are further increased to 221 Mb/s or 363 Mb/s and 6.8 mW or 10.9 mW of harvested energy by implementing a 2-by-2 or 4-by-4 OPV-based MIMO system, respectively, for the first time. The presented MIMO structure can soon become a reality considering the rapid advancements in optical device technologies and MIMO communication techniques, which would enable reliable, bidirectional, energy-efficient, and cost-effective OWC.

## Results

### OPVs

**Fig. 2:**
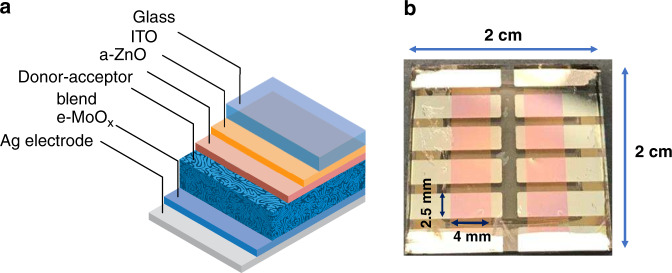
OPV design. **a** Schematic of the inverted OPV device architecture; ITO: indium tin oxide, a-ZnO: amorphous zinc oxide, e-MoOx: evaporated molybdenum oxide, Ag: element silver. **b** Photograph of the fabricated OPV sample. It shows eight individual cells (red squares) and four common ground pads at the corners.

OPVs were fabricated in the inverted configuration of ITO/ZnO/active layer/MoO_3_/Ag, as shown in Fig. 2a. The active layer was a BHJ of narrow bandgap donor polymers mixed with fullerene and nonfullerene acceptors. Although fullerenes are effective acceptors for charge separation in OPVs, their absorption in the visible light spectrum is poor. In the active layers of state-of-the-art highly efficient OPVs, the PC_71_BM fullerene acceptor is replaced by nonfullerene acceptors such as EH-IDTBR, with higher absorption in the visible range and thus higher efficiency^32^. The donor polymer in the present investigation was selected based on its high PCE and the high open-circuit voltage, *V*_oc_ = 1 V, under 1 Sun (100 mW/cm^2^) when blended with the nonfullerene acceptor used here. The narrow absorption band of OPVs with a high open-circuit voltage, *V*_oc_ > 1 V, is beneficial for indoor low-intensity lighting conditions, as the drop in *V*_oc_ with light intensity will be smaller than that for silicon solar cells^33–35^. In the present work, the simultaneous energy harvesting and data detection properties of traditional and state-of-the-art inverted OPVs constituted by both fullerene- and nonfullerene-based BHJs, such as PTB7:PC_71_BM, PTB7-Th:PC_71_BM, and PTB7-Th:EH-IDTBR, are investigated. The architecture of the OPV device structure is shown in Fig. 2a, and the actual fabricated solar cells are shown in Fig. 2b. There are eight individual solar cells (the red areas) on a 2 cm by 2 cm substrate and four common ground pads at the corners. The eight solar cells have separate contacts so that the output current from each of them is individually accessible. Therefore, the individual cells can be used as separate receivers, as in the current work, or their signals can be combined so that the whole panel acts as one receiver.

The characteristics of these solar cells were tested under both solar spectrum and indoor lighting illumination. The results are presented in the following subsections. The emission spectra of different illumination sources are demonstrated in Supplementary Information, Fig. S1.

### 1) OPV performance—solar spectrum illumination

**Fig. 3:**
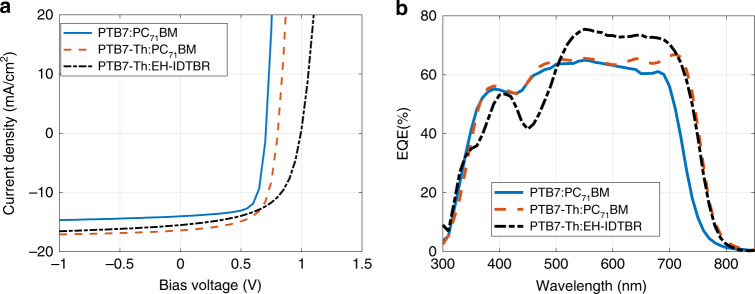
Performance of OPV cells under solar spectrum illumination. **a** *J–V* characteristics of OPV cells are shown corresponding to three different BHJ blends of PTB7:PC_71_BM, PTB7-Th:PC_71_BM and PTB7-Th:EH-IDTBR, measured under 100 mW/cm^2^ light intensity from a solar simulator. **b** EQE spectra of the corresponding OPV cells.

The absorption spectra of the three different OPV blend systems were measured and are presented in the Supplementary Information, Fig. S2. The absorption spectra of the PTB7:PC_71_BM and PTB7-Th:PC_71_BM blends span 350 nm–750 nm, whereas for the PTB7-Th:EH-IDTBR blend, a narrower absorption spectrum of 550 nm–750 nm in the visible light spectrum is observed. The PCEs of the OPV cells were determined from their current density-voltage (*J–V*) characteristics under 100 mW/cm^2^ light intensity from a solar simulator at a distance of 40 cm. The *J–V* characteristics and the external quantum efficiency (EQE) of each blend system are shown in Fig. 3, and the PV performance parameters are presented in the Supplementary Information, Table S1. PTB7:PC_71_BM demonstrates a PCE of ~7.6%, and the PTB7-Th-based BHJs exhibit a higher PCE of ~8.7%, which can be attributed to the narrower bandgap of PTB7-Th compared with PTB7, which is reflected in its larger short-circuit current density *J*_sc_. The PCE of the nonfullerene acceptor BHJ (PTB7-Th:EH-IDTBR) is ~8.8%, and it has a higher *V*_oc_∼1 V than the fullerene-based BHJ solar cells. The high *V*_oc_ of the OPVs with BHJs based on a nonfullerene acceptor can be attributed to the reduced energy loss via nonradiative recombination during exciton dissociation and generation of charge carriers^36–38^.

### 2) OPV performance—indoor lighting conditions

**Fig. 4:**
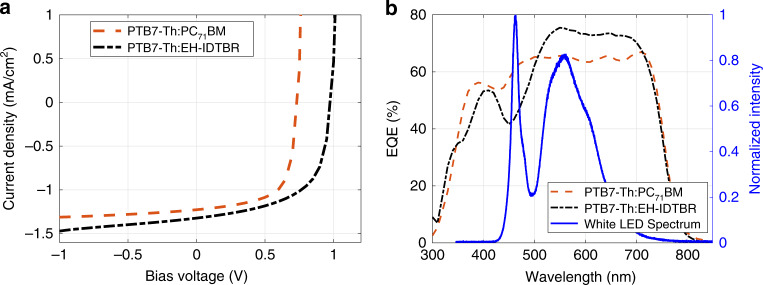
Performance of OPV cells under indoor lighting. **a** *J–V* characteristics of OPV cells are shown corresponding to BHJ blends of PTB7-Th:PC_71_BM and PTB7-Th:EH-IDTBR under white LED illumination. **b** Graph showing the spectral overlap of the EQE of the PTB7-Th:PC_71_BM and PTB7-Th:EH-IDTBR blends (left axis) with the spectrum of the white LED (right axis).

In addition to the performance evaluation of the PTB7-Th-based solar cells under 1 Sun intensity, their performance under indoor lighting conditions was also measured. Indoor lighting in residential buildings and offices is dominated by fluorescent lights and white LEDs, which are significantly different from the solar spectrum in intensity and spectral content. Under white LED lighting with an illumination intensity of 5.9 mW/cm^2^, the PCE is 11% for PTB7-Th:PC_71_BM with an output power of 0.64 mW/cm^2^ and 14% for PTB7-Th:EH-IDTBR with an output power of 0.83 mW/cm^2^ (Table S2). The corresponding *J–V* characteristics are shown in Fig. 4a. The higher PCE of the PTB7-Th:EH-IDTBR blend compared with PTB7-Th:PC_71_BM mainly originates from the higher *V*_oc_ and *J*_sc_ under indoor lighting conditions. This can be attributed to the higher EQE of the PTB7-Th:EH-IDTBR blend for the emission spectra of white LEDs, as shown in Fig. 4b. This reduces the carrier loss from thermalization and non-absorption. The PV performance of the PTB7-Th:EH-IDTBR blend under fluorescent lighting, with an illumination intensity of 0.7 mW/cm^2^, was also tested, and a PCE of 13.6% was obtained with an output power of 95 µW/cm^2^. A comparison of the PV properties of our best-performing OPV blend, PTB7-Th:EH-IDTBR, under 1 Sun and indoor illumination is shown in Fig. S4. Compared with 1 Sun illumination, the PCE is considerably improved under indoor illumination, and 80% of *V*_oc_ is retained along with a slightly improved fill factor. The demonstrated high PCE of the OPVs affirms their promising capability for indoor light energy-harvesting applications. In addition, the EQE spectra of commercial solar cells, such as silicon and GaAs, are compared with those of our OPVs in Supplementary Note 1.

### Communication protocols

The availability of a safe and affordable spectrum has become a crucial factor of wireless systems. Although there is a vast amount of free and safe spectrum in the visible light domain, the performance of visible light communication (VLC) is generally limited by the bandwidth of optical components, such as LEDs and PDs. The slow response of these optical components in electrical–optical signal conversion causes this bandwidth constraint. In an OPV-based system, the system bandwidth is limited by OPV cells to a few MHz. Therefore, it is important to optimize the system to obtain the highest possible data rate with this bandwidth constraint. As the system is designed based on single-sided baseband modulation, the maximum frequency used for signal modulation directly determines the achievable data rate of the system.

OFDM is an efficient signaling scheme by means of which a data rate close to the theoretical capacity limit of the communication channel can be achieved. It has been proven in practice that OFDM can deliver high data rate communication and, at the same time, provide relatively simple solutions for some of the most challenging problems in wireless communication, such as the frequency selectivity of the wireless channel that could cause severe intersymbol interference^39^. OFDM was first developed for RF communication, where signals are bipolar and complex. However, some modifications are necessary to make an OFDM signal real and positive, which is suitable for IM/DD systems in VLC^5,40^. In addition, by using adaptive bit and power loading, it is straightforward to maximize the data rate in a VLC system that usually exhibits a low-pass frequency-selective response^41^. The available signal-to-noise ratio (SNR) determines the modulation depth of each subcarrier at a specific target bit error ratio (BER), and as a result, the total achievable data rate can be estimated^42^.

Direct current biased optical OFDM (DCO-OFDM) is an efficient and low-complexity method used in VLC to achieve high data rates^6,12,40^. By imposing Hermitian symmetry on the signal vector in the frequency domain, a real-time domain signal is obtained after the Fourier transformation. Then, a constant direct current (DC) bias is added to the signal so that it becomes positive. However, depending on the value of the DC bias, a few samples may still be negative, which are clipped to zero before transmission. Taking into account the Hermitian symmetry and the DC bias (i.e., no data can be modulated in subcarriers X_0_ and $X_{N_{\mathrm{s}}/2}$), the frequency-domain subcarrier vector, **X**, is described as follows:

1 $${\mathbf{X}} = \left[ {0,X_1, \ldots ,X_{\frac{{N_{\mathrm{s}}}}{2} - 1},0,X_{\frac{{N_{\mathrm{s}}}}{2} - 1}^ \ast , \ldots ,X_1^ \ast } \right]$$

where *N*_s_ is the number of available subcarriers. In (1), $X_k,1 \le k \le \frac{{N_{\mathrm{s}}}}{2} - 1$, are the modulated data symbols at each subcarrier, *k*. Each subcarrier is modulated by an *M*_*k*_-ary quadrature amplitude modulation signal. The signal constellation for each subcarrier may be different, hence the use of the subscript *k*. The value of *M*_*k*_ depends on the available SNR at the respective subcarrier. In this paper, based on the adaptive bit loading algorithm^41^, the maximum *M*_*k*_ is found iteratively for the available SNR at each subcarrier so that the BER is below the target BER of 4.7 × 10^−3^. Error-free data transmission is then feasible using a forward error correction algorithm with 6.25% coding overhead^43^. More details are presented in Supplementary Note 2.

Signal multiplexing in the spatial domain is an effective solution to increase the achievable data rate in VLC systems with multiple inputs and multiple outputs. Space constitutes an extra degree of freedom in addition to the time and frequency dimensions. Spatial multiplexing is a MIMO technique and is particularly suitable for an OPV-based system since multiple cells are available at the receiver side to form a panel and the additional hardware complexity for MIMO implementation is minimal. The challenge is to manage and reduce the cross-talk between different links. The cross-talk results in an interference signal, which, similar to thermal noise, reduces the total system capacity. In an OPV-based system, interference in the optical domain can be eliminated almost completely by using appropriate lenses that allow light beam alignment to specific individual receiver cells. This is also referred to as imaging MIMO. However, interference in the electrical domain is unavoidable in practical scenarios because of the dual use of the OPV panel as an energy-harvesting device and a communication device. To be able to aggregate the energy of multiple OPV cells, they are usually connected either in series or in parallel. In other words, this is a one-port system. In contrast, it is necessary to extract signals from each OPV cell individually for MIMO operation. This requires a multi-port system. As a consequence, a circuit design is required that aggregates DC power while at the same time enabling extraction of alternating current (AC) signals from each OPV cell. This issue inevitably causes interference in the electrical domain. We note that while it is straightforward to improve the harvested energy by increasing the number of OPV cells, this poses an increasing challenge for interference mitigation for efficient MIMO communication.

### Proof-of-principle experiments for OPV-based communication

**Fig. 5:**
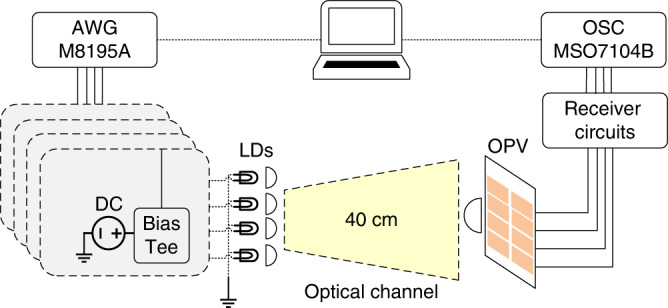
Block diagram of the system. A digital signal is generated by a computer and sent to an arbitrary waveform generator (AWG) for digital-to-analogue conversion. The analogue signal is combined with a direct current (DC) bias using a bias tee. Depending on the experiment format, one, two, or four laser diodes (LDs), mounted in lens tubes with an adjustable focal length, are used to transmit the optical signals. One large lens is used at the receiver to focus the light beams on the required number of OPV cells. The receiver circuit is used to extract the signal and harvest the DC power. Details of the receiver circuit are provided in Supplementary Information. An oscilloscope (OSC) is used to convert the analogue signal to a digital signal and send it to a computer for processing. More details of the setup are provided in the Materials and methods section.

In this section, experimental results are presented that demonstrate the feasibility of simultaneous high-speed data transmission and efficient energy harvesting by multiple OPVs. The communication performance of specially fabricated OPVs is investigated by the experimental setup shown in Fig. 5. A single light source and a single OPV cell are used for the SISO setup, whereas two and four of each are used for the 2-by-2 and 4-by-4 MIMO experiments, respectively. Random data generation and data detection from the received signal are processed offline by means of a computer using MATLAB software. The details of the setup shown in Fig. 5 are given in the Materials and methods section.

**Fig. 6:**
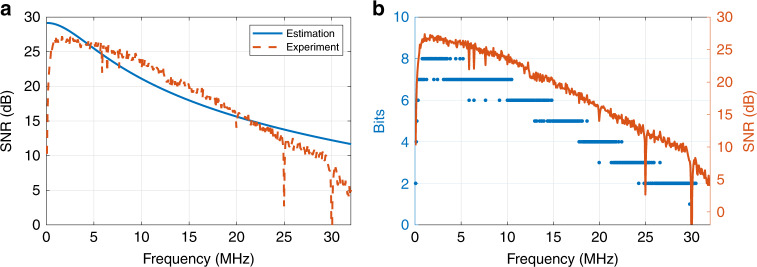
Communication performance of the PTB7-Th:EH-IDTBR blend OPV cell. **a** The estimated and measured SNR values are shown for the best single cell among the eight available cells of the OPV panel. **b** The number of bits loaded onto each subcarrier channel (left axis) and the measured SNR values (right axis). A greater number of bits is allocated to subcarriers that experience a higher available SNR. This is referred to as adaptive bit loading.

A summary of the results for the different types of OPVs is presented in Table 1. A data rate of *ρ* = 147.5 Mb/s and a harvested power of *E*_H_ = 3.7 mW are obtained using PTB7-Th:EH-IDTBR. The achieved data rate is the highest reported for a SISO setup when OPVs are used as receivers compared with previously published data rates using OPVs^26,27^. Estimated and measured SNR values are presented in Fig. 6a. Moreover, the bit loading results are shown in Fig. 6b. Note that these results correspond to the best single cell among the eight available cells of the OPV panel. The recorded BERs are below the hard-decision forward error correction coding threshold of 4.7 × 10^−3^, which means that with a 6.25% coding overhead, essentially error-free communication is possible^43^. The measured −3 dB bandwidths of the OPVs are 1.32 MHz, 1.26 MHz, and 2.77 MHz for PTB7:PC_71_BM, PTB7-Th:PC_71_BM, and PTB7-Th:EH-IDTBR, respectively. The channel responses of the OPVs are presented in the Supplementary Information, Fig. S6. A total of 512 OFDM subcarriers were used. Owing to the adaptive bit and power loading used, the modulation bandwidths for PTB7:PC_71_BM, PTB7-Th:PC_71_BM, and PTB7-Th:EH-IDTBR were 19.48 MHz, 17.52 MHz, and 30.1 MHz, respectively, which are considerably higher than the −3 dB bandwidths.

**Table 1:** Results for energy harvesting and data transmission for SISO experiments.

OPV blend	*E*_H_ [mW]	*ρ* [Mb/s]	BER
PTB7:PC_71_BM	3.3	90.3	3.6 × 10^−3^
PTB7-Th:PC_71_BM	3.5	78.4	1.6 × 10^−3^
PTB7-Th:EH-IDTBR	3.7	147.5	2.8 × 10^−3^

*E*_*H*_ Harvested power, *ρ* data rate.

Note that the light spectrum of the laser diode (LD) used in this experiment is part of the indoor white light emission spectrum. White light with the desired temperature can be produced by adding blue and green light sources. As a consequence, wavelength division multiplexing (WDM) can be implemented to enhance the data rates^12^. We focus on the single-color transmission here and leave the inclusion of WDM for future work. In the following, the MIMO system is presented to signify the potential of using OPVs as optical receivers for future VLC applications.

**Fig. 7:**
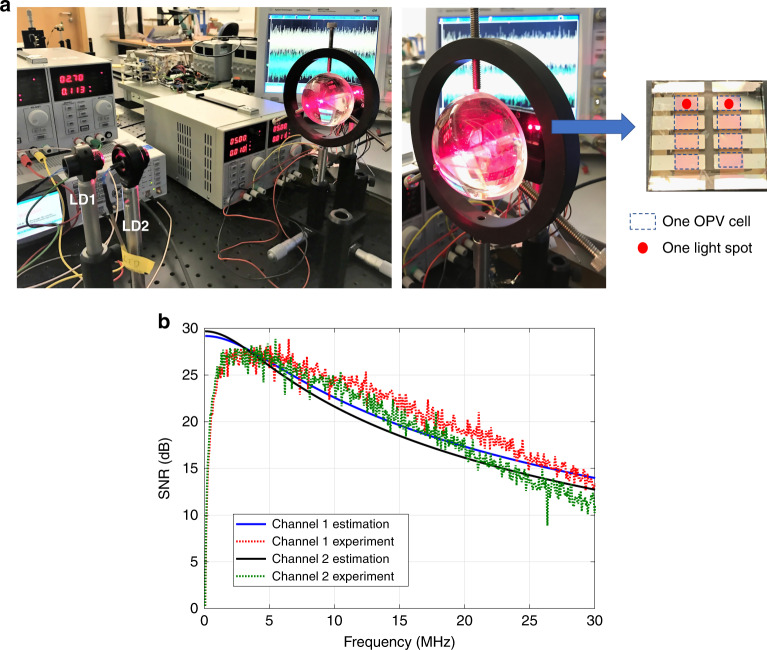
Proof-of-concept of MIMO experiment. **a** Photograph of the 2-by-2 MIMO setup in the lab (left). Two laser diodes (LDs) are used to transmit the signals. One large aspheric lens is used at the receiver to focus the light beams on two separate OPV cells (right). **b** Estimated and measured SNR values for two channels of the 2-by-2 MIMO experiment.

For the 2-by-2 MIMO setup, two equivalent LDs and two OPV cells on the same panel were used. The transmitters, LDs (Thorlabs, 6544FM) mounted in lens tubes with an adjustable focal length (Thorlabs, LT230220P-B, 5.6 mm diameter), were oriented towards the receiver lens. Light beams from the two LDs were each focused on one of the cells by a single lens (Thorlabs, ACL7560, 75 mm diameter). The distance between the receiver lens and the OPV panel was manually adjusted until two clear spots were observed on two separate OPV cells, and consequently, two parallel optical channels were generated in the spatial domain. This allowed us to have links with negligible optical cross-talk. A picture of the setup is shown in Fig. 7a, where the two light spots originating from two laser sources can be seen. The spot sizes were adjusted to fit within each OPV cell. The size of the OPV panel, which consists of eight cells, is 2 cm by 2 cm, and each cell is 4 mm by 2.5 mm in size; thus, the spot size is roughly a circle with a diameter of 2.5 mm. For the MIMO experiments, subsets of the eight OPV cells were chosen. Although the optical channel matrix was almost diagonal, the electrical channel and consequently the overall channel matrices were nondiagonal due to the structure of the receiver circuit. Details are presented in Supplementary Notes3 and 4. Therefore, there is some cross-talk in the electrical domain among the received signals from each OPV cell. To mitigate this cross-talk effect, zero forcing (ZF) was used to demultiplex the desired signal of each channel. ZF was performed by multiplying the received signal in vector format by the inverse of the channel matrix. Details are presented in the Methods section. The estimated and measured SNR values are demonstrated in Fig. 7b. Measurements were taken using PTB7-Th:EH-IDTBR OPVs. The results are shown in Table 2. The achieved data rates for the PTB7:PC_71_BM and PTB7-Th:EH-IDTBR OPVs are 122 Mb/s and 221 Mb/s, respectively, whereas the measured harvested powers are 5.8 mW and 6.8 mW, respectively. Note that the minimum mean square error algorithm was also used as an alternative demultiplexing technique. However, the results were almost the same due to the specific channel matrix values and relatively high SNRs at the utilized subcarriers. Therefore, only results using ZF are reported. A 4-by-4 MIMO experiment was also set up and tested using four red LDs and four OPV cells of type PTB7-Th:EH-IDTBR. The results are shown in Table 2. A record data rate of 363 Mb/s is achieved using four OPVs. We note that the capacity per channel decreases for a larger number of cells due to the increased interference.

**Table 2:** Results for energy harvesting and data transmission for MIMO.

OPV blend	MIMO	*E*_H_ [mW]	*ρ* [Mb/s]	BER
PTB7:PC_71_BM	2-by-2	5.8	122	3.5 × 10^−3^
PTB7-Th:EH-IDTBR	2-by-2	6.8	221	3.4 × 10^−3^
PTB7-Th:EH-IDTBR	4-by-4	10.9	363	1.1 × 10^−3^

*E*_H_ Harvested power, *ρ* data rate.

## Discussion

This paper reports a fundamental breakthrough in the capability of OPVs to function as both energy-harvesting devices and high-speed data detectors. We achieved this by jointly advancing PV materials, device structures, and communication techniques. The OPVs used in this work exhibit a −3 dB bandwidth as high as 2.77 MHz, which is the highest ever reported. Furthermore, the OPVs demonstrate a high power conversion efficiency of 13–14% under indoor lighting conditions. For PTB7-Th:EH-IDTBR-type OPVs, a data rate of 147.5 Mb/s (i.e., three times faster than the previous single OPV device) was achieved for a SISO system. We used a MIMO technique consisting of a maximum of four LDs and four OPVs to increase the data rate to a remarkable value of 363 Mb/s. With the four OPV cells, we simultaneously extracted a power of 10.9 mW.

Although the reported results are for a specific system configuration of four light transmitters and four OPV cells integrated into a single panel, the reported methodology is generally applicable. As a result, by using the proposed method, it is possible to trade the physical size of the system for both harvested energy and data rate.

OPVs are very attractive because they are easily made and can be flexible, thereby allowing their integration into internet-connected devices. In addition, compared with inorganic detectors, OPVs have the potential to be significantly cheaper, which is a key driver of their large-scale commercial adoption. Although the demand for wireless data transmission capacity is ever increasing, VLC provides unregulated, safe, and vast resources to alleviate emerging wireless capacity bottlenecks. The additional feature of being able to directly harvest the energy from the data-carrying signal could be used to enhance the battery life of mobile devices, which is one of the key issues of modern wearables.

## Materials and methods

### Fabrication and testing of organic solar cells

Inverted organic solar cells were fabricated on prepatterned ITO-coated glass. The ITO-coated glass substrates were cleaned in detergent (sodium dodecyl sulfate), successively ultrasonicated in deionized water, acetone, and isopropyl alcohol, and exposed to oxygen plasma for 3 min. PTB7:PC_71_BM blend solutions were prepared by dissolving the components in a ratio of 1:1.5 (by weight), with a total concentration of 25 mg/mL in chlorobenzene, with 3 vol% DIO. In the case of the PTB7-Th:PC_71_BM blend, the donor:acceptor components were weighed in a ratio of 1:1.5, with a total concentration of 25 mg/mL in ortho-dichlorobenzene solvent. For the PTB7-Th:EH-IDTBR blend system, the active layer solution was prepared by blending the donor-acceptor components in a 1:1 weight ratio, with a total concentration of 20 mg/mL in ortho-dichlorobenzene solvent. For all the inverted organic solar cells fabricated, the electron transporting layer was a thin film of amorphous ZnO (a-ZnO) with a thickness of ~25 nm and was prepared following the method used by Jagadamma et al.^44^. The active layer was prepared by spin coating on glass/ITO/a-ZnO substrates inside a nitrogen-filled glove box as follows: for the PTB7:PC_71_BM blend, the spin-coating condition was 1500 rpm for 60 s; for the PTB7-Th:PC_71_BM layer, spin coating was performed at 1200 rpm for 60 s; and for PTB7-Th:EH-IDTBR, the spin-coating process was carried out at 900 rpm for 60 s. The samples were then transferred to a vacuum thermal evaporator (1 × 10^−6^ mbar base pressure) and kept under vacuum overnight before thermally evaporating the hole transporting layer of MoOx (4 nm) and anode of Ag (100 nm) using a shadow mask. The aperture area of the shadow mask used for the measurement of the OPV devices was 0.065 cm^2^. After electrode deposition, the devices were encapsulated with a UV optical adhesive and a glass coverslip. The current–voltage characteristics were determined under an illumination intensity of 100 mW/cm^2^ in the air using an AM 1.5 global Sciencetech (SS150 -AAA) solar simulator at a distance of 40 cm and a Keithley 2400 source-measure unit. The illumination intensity was verified with a calibrated monosilicon detector and a KG-5 filter. The EQE measurements were performed at zero bias by illuminating the device with monochromatic light supplied from a xenon arc lamp in combination with a dual-grating monochromator. The number of photons incident on the sample was calculated for each wavelength by using a silicon photodiode calibrated by the National Physical Laboratory. For the indoor measurements, the LED light source used was a Cree XML T6, and the illumination intensity (5.9 mW/cm^2^) was adjusted by changing the input voltage at a distance of 4 cm. A fluorescent lamp (RS components, PL 11 lamp, 11 W) was also used as an illumination source (0.7 mW/cm^2^) at a distance of 30 cm. The irradiance level was measured using an RK 5710 power radiometer and an Optometer P9710. The absorption spectra of the active layer blends were recorded using a CARY 300 UV-Visible Spectrophotometer.

### SNR measurement and estimation

Prior to the data transmission, several training sequences were transmitted by each light source, one at a time for the MIMO case. A sequence of known quadrature phase-shift keying-modulated symbols was used in a DCO-OFDM transmission scheme. This allowed for accurate estimation of the frequency response as well as the channel gain at each subcarrier. First, channel gains at different frequencies were measured and averaged over 80 OFDM frames. Then, the noise power was estimated as the difference between the total received power and the noise-free received signal power, which was determined based on the measured channel gain and the known training sequence. For the SISO case, the SNR was estimated as the ratio of the noise-free received signal power and noise power. Note that the sample sequence generated in MATLAB software was assumed to be the input signal, and the sample sequence captured by the oscilloscope after the digital-to-analog conversion was assumed to be the output signal. Therefore, the total channel response, including the effects of all components, was determined.

For the MIMO scenario, the communication channel at the *k*^th^ subcarrier is an additive white Gaussian noise channel. The system model for *N*_t_ transmitters and *N*_r_ receivers can therefore be written as follows:

2 $${\mathbf{y}}_k^{N_{\mathrm{r}} \times 1} = {\mathbf{H}}_k^{N_{\mathrm{r}} \times N_{\mathrm{t}}}{\mathbf{x}}_k^{N_{\mathrm{t}} \times 1} + {\mathbf{n}}_k^{N_{\mathrm{r}} \times 1}$$

where vectors ${\mathbf{y}}_k^{N_{\mathrm{r}} \times 1}$ and ${\mathbf{n}}_k^{N_{\mathrm{r}} \times 1}$ are the received signal and noise, respectively. The channel matrix ${\mathbf{H}}_k^{N_{\mathrm{r}} \times N_{\mathrm{t}}} = {\mathbf{G}}_k^{N_{\mathrm{r}} \times N_{\mathrm{r}}}{\mathbf{H}}_0^{N_{\mathrm{r}} \times N_{\mathrm{t}}}$ is the total channel matrix at the corresponding subcarrier, where the matrices ${\mathbf{G}}_k^{N_{\mathrm{r}} \times N_{\mathrm{r}}}$ and ${\mathbf{H}}_0^{N_{\mathrm{r}} \times N_{\mathrm{t}}}$ represent the electrical and optical channel gains, respectively. The vector ${\mathbf{x}}_k^{N_{\mathrm{t}} \times 1}$ refers to the transmitted signal at the *k*^th^ subcarrier. Elements of the channel matrix are estimated as previously described and can be calculated using existing models^28^. The effective noise consists of thermal noise and shot noise and is assumed to be white Gaussian ${\cal{N}}(0,{\it{\upsigma }}^2{\mathbf{I}}_{N_{\mathrm{r}}})$, where ${\mathbf{I}}_{N_{\mathrm{r}}}$ is an ideal matrix of size *N*_r_. To remove the cross-talk between channels, ZF was applied by multiplying the received vector by the inverse of the channel matrix ${\mathbf{y}}_{\it{k}}{\mathbf{H}}_k^{ - 1}$. Then, the SNR was estimated in the same way as described for SISO. The SNR at the *k*^th^ subcarrier can also be calculated as^43^

3 $$\gamma _{k,n}^{{\mathrm{ZF}}} = \frac{{P_{{\mathrm{elec}}}^k}}{{\sigma ^2\left[ {\left\{ {{\mathbf{H}}_k^\dagger {\mathbf{H}}_k} \right\}^{ - 1}} \right]_{nn}}}$$

where $P_{{\mathrm{elec}}}^k$ is the total electrical input signal power at the *k*^th^ subcarrier, **†** denotes the matrix conjugate transpose, and []_*nn*_ denotes the *n*^th^ diagonal element.

### Data transmission measurement

A red LD (Thorlabs, HL6544FM) with a dominant wavelength of 660 nm was used as the light source. The DC bias was provided by the bench power supply (TENMA 72-10505). The OFDM signal was generated by a laptop using MATLAB and fed to the LD by an arbitrary waveform generator (AWG, Keysight M8195A). The OFDM signal and DC bias were combined with a bias tee (Mini-Circuit, ZFBT-4R2GW+). Each transmitter (Thorlabs, 6544FM) was mounted in a lens tube with an adjustable focal length (Thorlabs, LT230220P-B, 5.6 mm diameter). For the 2-by-2 MIMO setup, the two LD modules were arranged side by side with a distance of 2.5 cm between them. At the receiver, a single aspheric condenser lens (Thorlabs, ACL7560, 75 mm diameter) was used. The link distance was set to 40 cm. On the receiver end, the fabricated OPV panels shown in Fig. 2 were used, and the output from each OPV cell was connected to a custom-designed receiver circuit that contained two branches for signal detection and energy harvesting. A capacitor and a load resistor (50 Ω) were incorporated into the signal detection branch of the circuit. The energy-harvesting branch contained an inductor and another load resistor (50 Ω). The LD was driven at 100 mA, and the measured output optical power was 56.2 mW. The received optical power was 49.6 mW. These powers were measured by an optical power meter (Thorlabs, S121C). Details of the circuit model for simultaneous energy harvesting and data transmission can be found in the Supplementary Information. The signal was captured by an oscilloscope (Keysight, MSO7104B) and sent to the laptop to be processed in MATLAB. The SNR was measured based on a training sequence, and for comparison, it was also theoretically approximated based on the estimated optical channel gain, noise power, and OPV parameters (capacitance, shunt resistance, etc.). Adaptive bit and power loading, as explained in the previous section, was used to determine the maximum modulation depth at each subcarrier. A sequence of random data bits was transmitted, and the BER was measured. The power dissipated by the load resistor in the energy-harvesting branch was regarded as the harvested power, which could be obtained from the voltage across the resistor and the current flowing through it.

## Supplementary information

Supplemental Material

## Data Availability

The authors declare that all data supporting the findings of this study are available within the paper and its supplementary information files. The source data underlying Figs. 3, 4, 6, and 7b of the main paper and supplementary Figs. S1–S4 and S6–S8 are provided as source data files at 10.15129/01bf34cf-889a-4f8c-b1ad-8bf482443228.
