# Supplementary material for: Organic photovoltaics for simultaneous energy harvesting and high-speed MIMO optical wireless communications
Source: Light Sci Appl. 2021 Feb 23;10:41. doi: 10.1038/s41377-021-00487-9 (PMC7902835; doi:10.1038/s41377-021-00487-9)
Supplement: Supplementary file 1 — Supplemental Material [file 41377_2021_487_MOESM1_ESM.pdf]

Organic Photovoltaics for Simultaneous Energy Harvesting and High-Speed MIMO Optical Wireless Communications

Iman Tavakkolnia[1+], Lethy K. Jagadamma[2+], Rui Bian[3+], Pavlos P. Manousiadis[2], Stefan Videv[1], Graham A. Turnbull[2], Ifor D. W. Samuel[2], Harald Haas[1]

[1]LiFi Research and Development Centre, Department of Electronic and Electrical Engineering, The University of Strathclyde, Technology Innovation Centre, 99 George Street, Glasgow, G1 1RD, UK
[2]Organic Semiconductor Centre, SUPA, School of Physics and Astronomy, University of St Andrews, St Andrews, KY16 9SS, UK.
[3]pureLiFi, Rosebery House, 9 Haymarket Terrace, Edinburgh, EH12 5EZ, UK.
[+]These authors contributed equally to this work.
[*]Corresponding author: Harald Haas, Ifor D. W. Samuel, Graham A. Turnbull. e-mails: harald.haas@strath.ac.uk, idws@st-andrews.ac.uk, gat@st-andrews.ac.uk .

Supplementary Information

Supplementary Figures and Tables

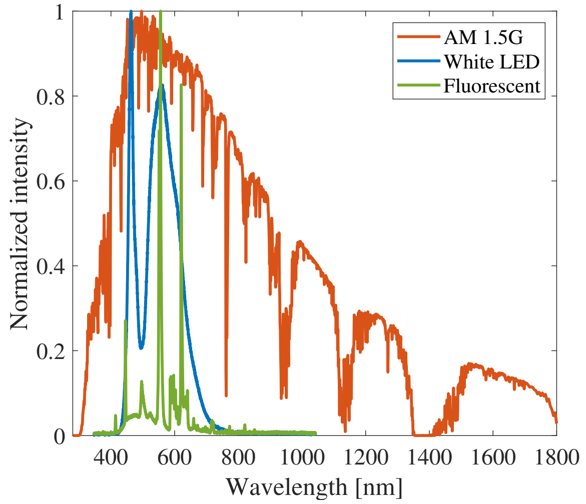

Figure S1: Comparison of indoor lighting spectra (from white LED and fluorescent lamp) with the AM 1.5 G solar spectrum.

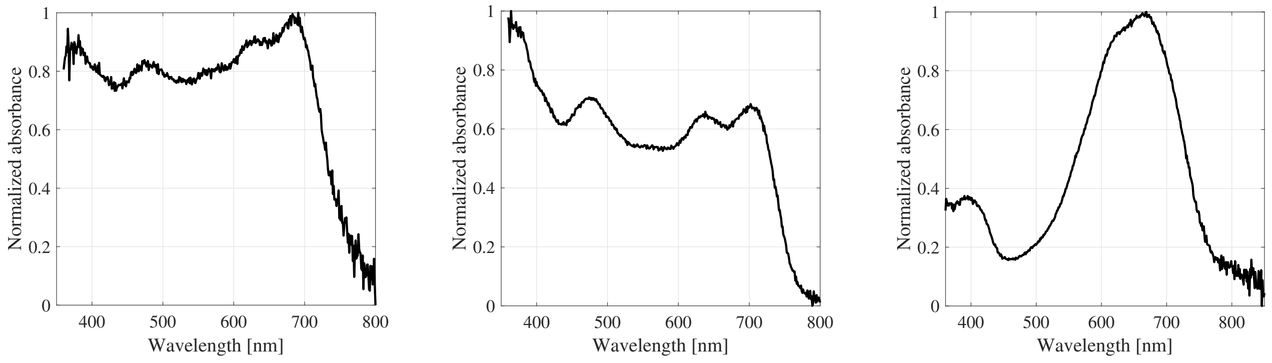

Figure S2: Absorption spectra of the three OPV active layer blends used in this study (a) PTB7:$PC_{71}BM$, (b) PTB7-Th:$PC_{71}BM$, (c) PTB7-Th:EH-IDTBR.

Table S1: Solar cell performance parameters of the three OPV active layer blends used in the present study under solar spectrum. (FF: fill factor)

	J_{sc} [mA/cm^2]	V_{oc} [V]	FF [%]	R_S [Ω.cm^2]	R_{SH} [Ω.cm^2]	PCE Average [%]	PCE Best [%]
PTB7:$PC_{71}BM$	14.2 ± 0.22	0.708 ± 0.004	72.9 ± 0.64	0.99 ± 0.22	1477 ± 403	7.34±0.14	7.58
PTB7-Th:$PC_{71}BM$	15.7 ± 0.69	0.793 ± 0.009	64.9 ± 0.82	1.23 ± 0.23	899 ± 139	8.06 ±0.36	8.66
PTB7-Th:EH-IDTBR	15.4 ± 0.28	0.997 ± 0.004	55.3 ± 1.93	1.73 ± 0.32	576 ± 102	7.82 ±0.27	8.79

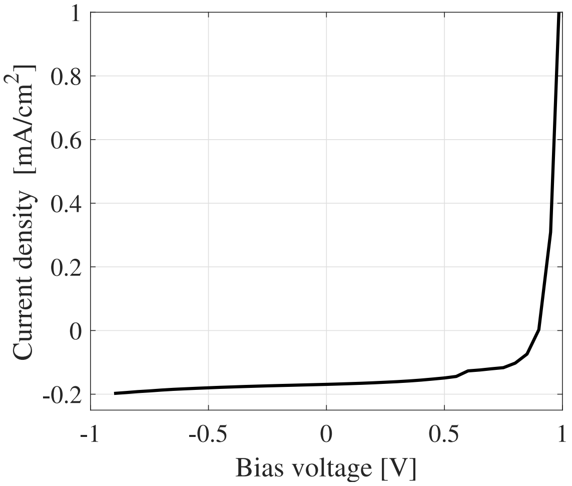

Figure S3: J-V characteristics of the PTB7-Th:EH-IDTBR blend under fluorescent illumination intensity of 0.7 mW/cm^2

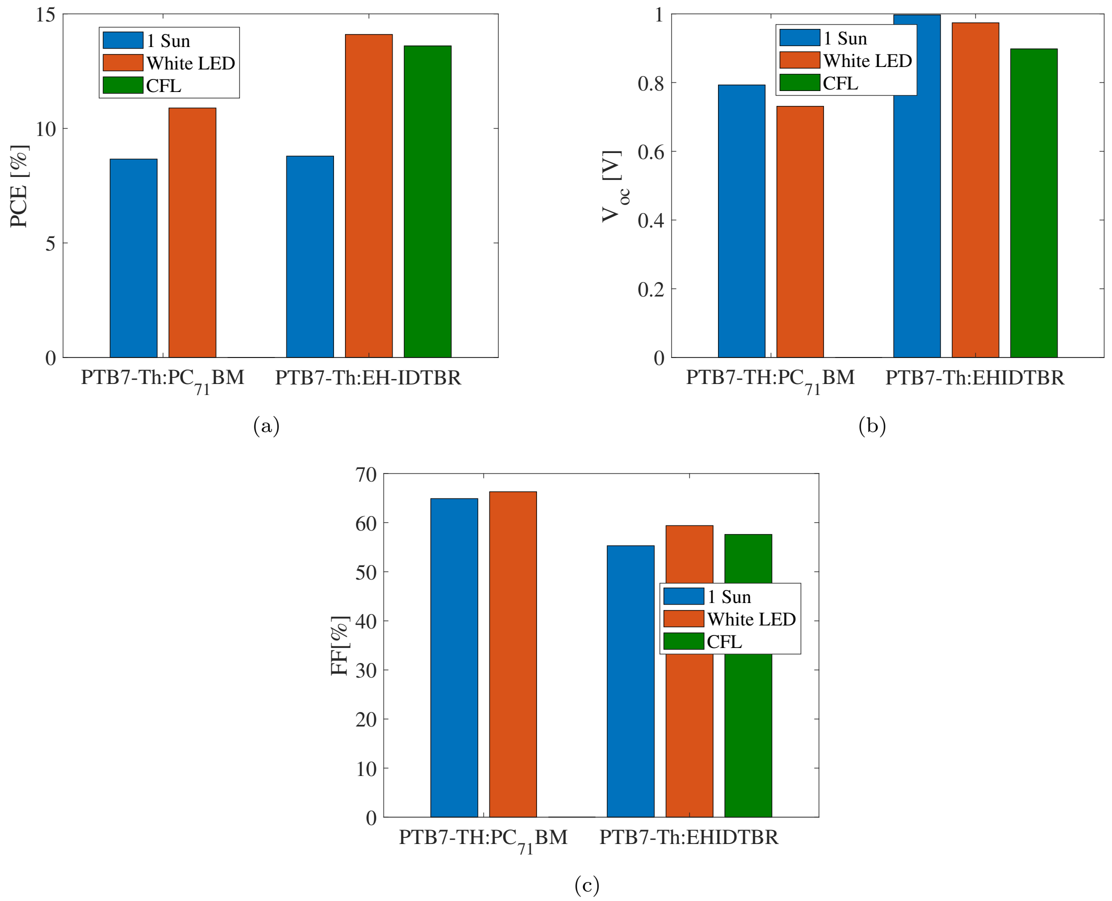

Figure S4: Comparison of the (a) power conversion efficiency (PCE) and photovoltaic performance parameters (b) open circuit voltage (V_{oc}) and (c) fill factor (FF) of the best performing OPV blend of PTB7-Th:EH-IDTBR under different illumination of 1 Sun, white LED and compact fluorescent lamp (CFL).

Table S2: Photovoltaic performance parameters of the PTB7-Th:$PC_{71}BM$ and PTB7-Th:EH-IDTBR solar cells as a function of indoor lighting condition.

	Light Source/Power [mW/cm^2]	J_{sc} [mA/cm^2]	V_{oc} [V]	FF [%]	PCE [%]	Output Power [mW/cm^2]
PTB7-Th:$PC_{71}BM$	White LED (5.9)	1.32	0.731	66.3	10.9	0.64
PTB7-Th:EH-IDTBR	White LED (5.9)	1.43	0.974	59.4	14.1	0.83
PTB7-Th:EH-IDTBR	CFL(0.7)	0.183	0.898	57.6	13.6	0.095

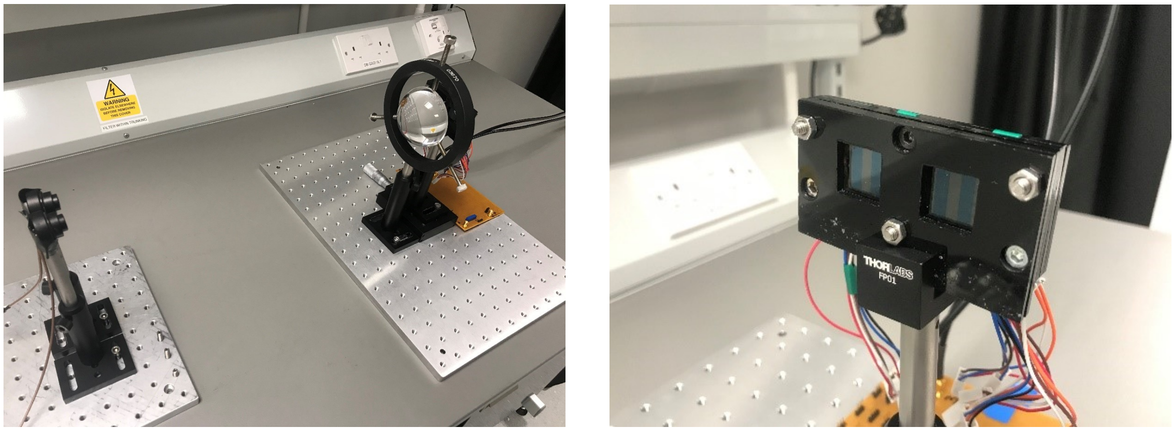

(a) (b)

Figure S5: (a) Transmitter laser diodes and receiver lens , (b) OPV holder (each can hold two panels).

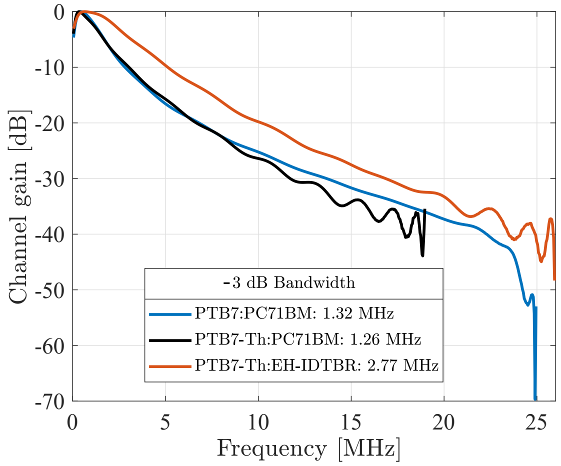

Figure S6: Frequency responses of SISO setup for different OPV types.

Supplementary Note 1: Comparing OPVs with commercial solar cells

The external quantum efficiency spectra of commercial solar cells, such as silicon and GaAs [1, 2], are compared with our OPVs in Fig. S8. The emission spectra of the indoor light sources, i.e., white LED and fluorescent lamp, are also shown for comparison. Whilst all the materials in Fig. S8 absorb fluorescent or LED light effectively, OPVs have a wider bandgap than the inorganic semiconductors and hence smaller thermalization losses. It has been reported previously [3, Fig. 3] that amorphous (a-Si) and crystalline silicon (c-Si) solar cells show only a marginal increase in power conversion efficiency under indoor lighting conditions compared to OPVs as shown in Fig S3. Table S3 shows the comparison of the photovoltaic properties of the OPV blend in our study and the silicon solar cell for different illumination levels [4, 5].

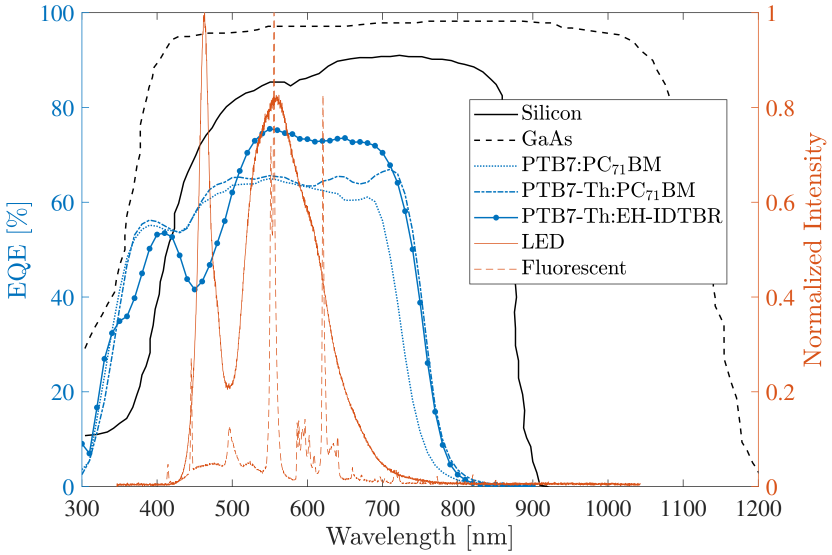

Figure S7: Comparison of OPVs with commercial solar cells (data taken from [1, 2]).

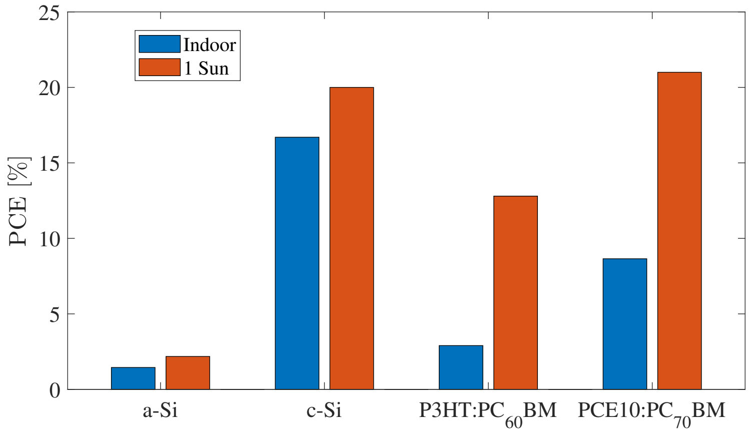

Figure S8: The PCE of the silicon solar cells compared to OPVs under 1 Sun and indoor lights (data taken from [3]).

	Illumination	Jsc [mA/cm^2]	V_{oc} [V]	FF [%]	PCE Avg [%]	PCE best [%]
PTB7-Th:EH-IDTBR	1 Sun 100 mW/cm^2	15.4±0.3	0.98±0.0	55.3±1.9	7.8±0.3	8.8
PTB7-Th:EH-IDTBR	Fluorescent 0.3 mW/cm^2	0.09±0.0	0.81±0.02	54.9±5.2	13.7±1.2	14.6
c-Si	1 Sun 100 mW/cm^2	19.2	0.52	70	7.0	
c-Si	Solar spectrum 0.1 mW/cm^2	0.167	0.15	40	1.0	

Table S3: The comparison of the photovoltaic properties of the OPV blend in our study and the silicon solar cell for different illumination levels [4, 5].

Supplementary Note 2: DCO-OFDM with adaptive bit loading

Orthogonal frequency division multiplexing (OFDM) is a multicarrier modulation technique with a long history in wireless communication systems. OFDM has been commercialized and incorporated in the latest generations of radio frequency (RF) wireless communication and many standards, such as the IEEE 802.11 family used in Wi-Fi. OFDM elegantly addresses the issue of inter-symbol interference which is particularly severe in high data rate wireless systems. This is because of the unavoidable multipath propagation. The available system bandwidth is divided into a number of orthogonal subchannels of equal bandwidth. Data is modulated on these subchannels. Due to the relatively small bandwidth of a subchannel compared to the system bandwidth, the frequency response per subchannel is generally flat if a cyclic prefix is added to the time-domain OFDM symbol. The cyclic prefix should be at least as long as the maximum multipath delay. This means that a single tap equalizer can be deployed in the frequency domain to equalize the channel. As a result, intersymbol interference is effectively mitigated.

Among the advantages of VLC is the possibility of implementation using low-cost commercially available light sources. This is realized by modulating the output optical power of the light source (i.e., intensity of light) by changing its driving current. Therefore, the time domain signal should only contain real and positive samples. Single carrier modulation schemes such as pulse amplitude modulation (PAM) can be easily adopted for VLC. However, OFDM generally produces complex-valued bipolar time-domain samples, and thus, it must be modified to be applicable to VLC. This can be achieved by using a property of the Fourier transform. Specifically, by imposing the Hermitian symmetry on the signal in the frequency domain, the time domain signal would be real. Subsequently, a constant direct current (DC) bias is added to the signal so that the signal becomes mostly positive. However, depending on the value of the DC bias a few samples may still be negative which need to be clipped to zero before transmission. Taking into account the Hermitian symmetry and the DC bias, the frequency domain subcarrier vector, $\mathbf{X}$, is described as

$$\mathbf{X} = [0, X_1, \cdots, X_{\frac{N_s-1}{2}}, 0, X^*_{\frac{N_s-1}{2}}, \cdots, X^*_1], \tag{1}$$

where N_s is the number of available subcarriers.

Each subcarrier is modulated by a M_k-ary quadrature amplitude modulation (M_k-QAM) symbol. The value of M_k depends on the available signal-to-noise ratio (SNR) at that subcarrier, γ_k, to meet the bit error ratio (BER) constraint, 4.7×10^{-3} in this paper. This technique is referred to as the adaptive bit loading. The use of adaptive bit loading leads to the maximum achievable data rate [7]. The BER at the kth subcarrier is approximated as follows [8]:

$$\text{BER}_k \simeq \frac{4\left(1 - \frac{1}{\sqrt{M_k}}\right)}{\log_2 M_k} \times \sum_{l=1}^{\min(2,\sqrt{M_k})} \text{Q}\left((2l-1)\sqrt{\frac{3\gamma_k}{2\left(M_k - 1\right)}}\right) \tag{2}$$

where Q(.) is the Gaussian Q-function. An algorithm is used for bit loading to determine the maximum M_k for $k = 2, \cdots, N_s/2 - 1$. At each subcarrier, the BER_k is calculated for the available SNR, γ_k, for values of M_k increasing from $M_k = 2$. The maximum value of M_k is found so that the BER_k is still below the target.

The overall data rate is given by [9]:

$$\rho = \frac{\sum_{k=2}^{\frac{N_s}{2}-1} \log_2 M_k}{(N_s + N_{CP})/2B}, \tag{3}$$

where B is the single-sided system bandwidth, and N_{CP} is the cyclic prefix size.

Supplementary Note 3: OPV model

A solar cell can be modeled by the circuit shown in Fig. S9 for simultaneous energy harvesting and communication analysis [6]. The photo-current source contains both the DC component I_{PH} and the AC component i_{PH}. The resistor r represents the small signal equivalent of the solar cell diode in the DC model. A capacitor C is used in parallel with R_{SH} to model the internal capacitance of the solar panel. An inductor L is set in series with R_S to model the inductance of wire connections within the solar panel. The output of the solar panel is connected to two branches: the communication branch and the energy harvesting branch. In the communication branch, a capacitor C_0 is connected to the output of the solar panel to block the DC signal and to pass the AC signal. The energy harvesting branch includes an AC signal filtering inductor L_0 and a load resistor R_L. The electrical power gain of the circuit can be determined as follows [6]:

$$\left|\frac{v(\omega)}{i_{PH}(\omega)}\right|^2 = \left|\frac{\frac{R_{LC}}{R_S + j\omega L + R_{LC}} \frac{R_C}{\frac{1}{j\omega C_0} + R_C}}{\frac{1}{r} + \frac{1}{\frac{1}{j\omega C}} + \frac{1}{R_{SH}} + \frac{1}{R_S + j\omega L + R_{LC}}}\right|^2 \tag{4}$$

where $\omega = 2\pi f$ is the angular frequency, $j = \sqrt{-1}$ and R_{LC} is the equivalent resistance of the two parallel branches and is calculated as follows:

$$R_{LC} = \frac{1}{\frac{1}{j\omega L_0 + R_L} + \frac{1}{\frac{1}{j\omega C_0} + R_C}} \tag{5}$$

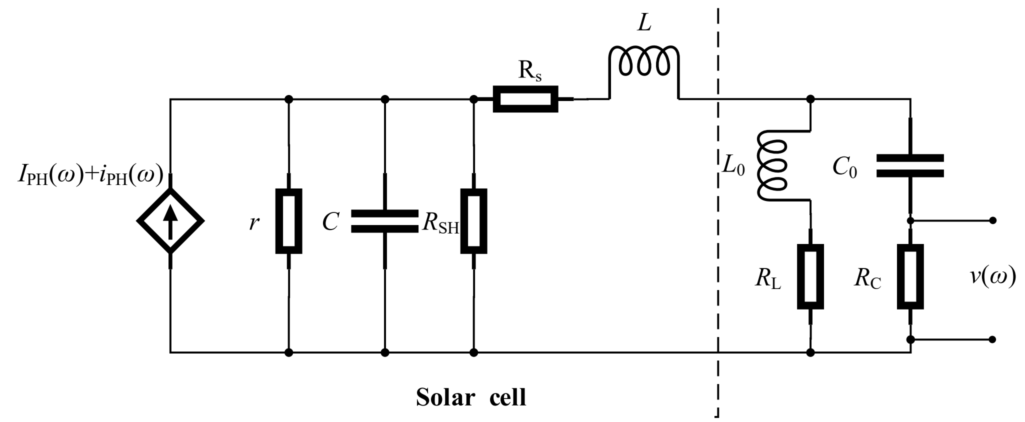

Figure S9: Solar panel model for simultaneous energy harvesting and data communication.

Supplementary Note 4: Electrical gain coefficient calculation

The electrical gain values can be derived from the RLC circuit which is formed by connecting multiple circuits shown in Fig. S9. For instance, as shown in Fig. S10 for a 2-by-2 MIMO system, each OPV cell requires a RC circuit for signal extraction while all OPVs are connected in series with a single RL circuit for energy harvesting. Considering a setup with N_{cell} OPV cells being connected in series, the elements of the electrical channel gain matrix, $\mathbf{G}^{N_r \times N_r} = [g_{nm}]^{N_r \times N_r}$, at the main diagonal are expressed as:

$$g_{(n=m)} = \frac{(N_{cell} - 1)R_x + R_{1,L}}{(N_{cell} - 1)R_x + R_{1,L} + R_{1,c}} \times \frac{R_0}{R_{1,s} + ((N_{cell} - 1)R_x + R_{1,L})//R_{1,c} + R_0} \times R_c, \tag{6}$$

The nondiagonal elements, i.e., the crosstalk gains, are nonzero and are expressed as:

$$g_{(n\neq m)} = \frac{R_0 + R_{1,\mathrm{s}}}{R_{1,\mathrm{c}} + R_0 + R_{1,\mathrm{s}}} \times \frac{R_{1,\mathrm{c}}}{(N_{\mathrm{cell}}-1)R_{\mathrm{x}} + R_{1,\mathrm{L}} + R_{1,\mathrm{c}}} \times \frac{R_0}{R_{1,\mathrm{s}} + ((N_{\mathrm{cell}}-1)R_{\mathrm{x}} + R_{1,\mathrm{L}})//R_{1,\mathrm{c}} + R_0} \times R_{\mathrm{c}}, \tag{7}$$

where // denotes connected in parallel, and: $R_{1,\mathrm{L}} = R_{\mathrm{L}} + j\omega L_0$, $R_{1,\mathrm{c}} = R_{\mathrm{C}} + \frac{1}{j\omega C_0}$, $R_{1,\mathrm{s}} = R_{\mathrm{s}} + j\omega L$, $R_0 = r//R_{\mathrm{SH}}//\frac{1}{j\omega C}$, and $R_{\mathrm{x}} = (R_0 + R_{1,\mathrm{s}})//R_{1,\mathrm{C_0}}$.

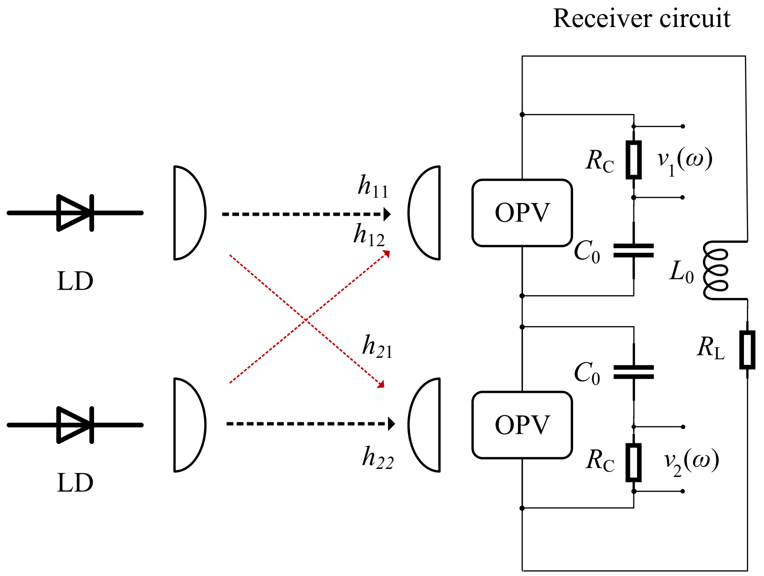

Figure S10: Solar panel model for simultaneous energy harvesting and data communication for a 2-by-2 MIMO system. $h_{n_\mathrm{r}n_\mathrm{t}}$ denotes the optical channel gain from the $n_\mathrm{t}^{\mathrm{th}}$ transmitter to the $n_\mathrm{r}^{\mathrm{th}}$ receiver. $v_1(\omega)$ and $v_2(\omega)$ represent extracted AC signals for the two data streams. Laser diode (LD).

References

[1] Moon, S. *et al.* Highly efficient single-junction GaAs thin-film solar cell on flexible substrate. *Scientific reports* **6**, 30107 (2016).

[2] Minnaert, B. and Veelaert, P. A proposal for typical artificial light sources for the characterization of indoor photovoltaic applications. *Energies* **7**, 1500-1516 (2014).

[3] Cutting, C.L., Bag, M. and Venkataraman, D. Indoor light recycling: a new home for organic photovoltaics. J *Journal of Materials Chemistry C* **4**, 10367-10370 (2016).

[4] Itaru, R. *et al.* Characteristics of Perovskite Solar Cells under Low-Illuminance Conditions. *Journal of Physical Chemistry C* **120**, 18986-18990 (2016).

[5] Reich, N.V. *et al.* Crystalline silicon cell performance at low light intensities. *Solar Energy Materials and Solar Cells* **93**, 1471-1481 (2009).

[6] Wang, Z. *et al.* On the Design of a Solar-Panel Receiver for Optical Wireless Communications with Simultaneous Energy Harvesting. *IEEE J. Sel. Areas Commun.* **33**, 1612–1623 (2015).

[7] Levin, H. E. A complete and optimal data allocation method for practical discrete multitone systems. Proceedings of 2001 IEEE Global Telecommunications Conference (GLOBECOM). San Antonio, TX, USA: IEEE, 2001, 369-374. .

[8] Narmanlioglu, O. *et al.* Link Adaptation for MIMO OFDM Visible Light Communication Systems. *IEEE Access* **5**, 26006-26014 (2017).

[9] Tsonev, D., Videv, S. & Haas, H. Unlocking spectral efficiency in intensity modulation and direct detection systems. *IEEE J. Sel. Areas Commun.* **33**, 1758–1770 (2015).
